# Unlocking the Potentials of Large Language Models in Orthodontics: A Scoping Review

**DOI:** 10.3390/bioengineering11111145

**Published:** 2024-11-13

**Authors:** Jie Zheng, Xiaoqian Ding, Jingya Jane Pu, Sze Man Chung, Qi Yong H. Ai, Kuo Feng Hung, Zhiyi Shan

**Affiliations:** 1 Department of Biomedical Sciences, City University of Hong Kong, Hong Kong, China; jayzhenglion@gmail.com; 2 Division of Paediatric Dentistry and Orthodontics, Faculty of Dentistry, The University of Hong Kong, Hong Kong, China; xiaoqiankq@connect.hku.hk (X.D.); rebecca.chungsm@gmail.com (S.M.C.); 3 Division of Oral and Maxillofacial Surgery, Faculty of Dentistry, The University of Hong Kong, Hong Kong, China; drjanepu@hku.hk; 4 Department of Health Technology and Informatics, The Hong Kong Polytechnic University, Hong Kong, China; hemis.ai@polyu.edu.hk; 5 Applied Oral Science & Community Dental Care, Faculty of Dentistry, The University of Hong Kong, Hong Kong, China

**Keywords:** large language models, orthodontics, scoping review, generative AI, LLMs, Chatbot, ChatGPT, GPT, artificial intelligence

## Abstract

(1) Background: In recent years, large language models (LLMs) such as ChatGPT have gained significant attention in various fields, including dentistry. This scoping review aims to examine the current applications and explore potential uses of LLMs in the orthodontic domain, shedding light on how they might improve dental healthcare. (2) Methods: We carried out a comprehensive search in five electronic databases, namely PubMed, Scopus, Embase, ProQuest and Web of Science. Two authors independently screened articles and performed data extraction according to the eligibility criteria, following the PRISMA-ScR guideline. The main findings from the included articles were synthesized and analyzed in a narrative way. (3) Results: A total of 706 articles were searched, and 12 papers were eventually included. The applications of LLMs include improving diagnostic and treatment efficiency in orthodontics as well as enhancing communication with patients. (4) Conclusions: There is emerging research in countries worldwide on the use of LLMs in orthodontics, suggesting an upward trend in their acceptance within this field. However, the potential application of LLMs remains in its early stage, with a noticeable lack of extensive studies and tailored products to address specific clinical needs.

## 1. Introduction

As a sub-branch of computer science, the study of artificial intelligence (AI) began as early as 1943 [1]. However, it was first introduced using the term “artificial intelligence” in 1956 [2]. AI is a broad field encompassing various computer programs designed to imitate human learning and reasoning, as well as the application of technologies like machine learning, deep learning, and natural language processing. It is gradually becoming capable of performing tasks that traditionally require human intelligence, significantly improving work efficiency [3,4,5]. With the development of artificial intelligence, significant changes have emerged across various fields. In finance [6], manufacturing [7], and transportation [8,9], AI has played a significant role in enhancing efficiency and driving innovation and progress within these areas. Similarly, in the medical field, the applications of AI are extensive and diverse. In the 1970s, the development of the MYCIN system marked a significant advancement in using AI for diagnosing bacterial infections and recommending antibiotic treatments. Since then, a growing number of studies have used AI to analyze medical images and manage electronic medical records [10,11,12,13]. In recent years, the maturation of AI technologies such as machine learning, deep learning, and others has further promoted the development of the medical field [14,15,16,17,18].

LLMs are a type of advanced AI model that depend on neural network architectures and deep learning techniques. They undergo pre-training and fine-tuning with large-scale text data, including articles and internet content, allowing them to perform a diversity of natural language processing tasks like text generation, question answering and others [19,20,21]. LLMs have demonstrated remarkable abilities in understanding human language and generating human-like dialogue, leading to their widespread use [22,23]. Some of these models, such as GPT-4 (OpenAI, 2023), Bard (Google, 2023), and Llama (Meta AI, 2023), are generative AI applications that generate human-like language texts and responses based on LLMs, which have been applied in a broad range of situations [24,25,26,27]. Although these models have presented notable potential in dialogue and question-answering tasks, their content accuracy and ethical issues still require strict supervision and management [28,29]. Moreover, the financial, educational, and medical sectors are increasingly adopting LLM-based applications, such as virtual tutors and automated medical documentation [30,31].

Numerous studies have shown that LLMs and their related software are widely used in the medical field. For example, LLMs can be employed for routine communication with patients to provide relatively professional responses that enhance patient engagement and satisfaction [32]. They also assist doctors in analyzing large amounts of clinical data to support decision making and improve overall healthcare outcomes [33]. Additionally, LLMs play an important role in the education of medical students by enhancing learning efficiency through personalized and interactive educational tools [34,35,36]. Although LLMs have been widely used in many medical fields, their research and application in orthodontics remain relatively limited. Therefore, there is still a lack of comprehensive understanding of their use in clinical practice and scientific research in orthodontics.

As a specialized branch of dentistry, orthodontics treatment is extensively utilized in clinical practice. The work in [37] indicates that malocclusion is currently considered one of the significant factors affecting oral health, with a highly variable prevalence among children and adolescents, estimated to range from 39% to 93%. However, orthodontic treatment provides notable intervention effects in correcting this condition [38], and in addressing certain facial asymmetries [39]. Orthodontics primarily concentrates on diagnosing, preventing, and correcting misaligned teeth and jaws, so it faces unique challenges and requirements that are different from other medical fields. In terms of diagnosis, orthodontics needs to combine clinical examinations with various imaging devices to comprehensively analyze the relationships between teeth, bones, and soft tissues. Additionally, orthodontic treatment plans are highly individualized, not only according to the specific situation of patients but also considering the long treatment period and the patient’s psychological factors [40,41,42]. LLMs offer substantial potential to enhance patient comprehension of the treatment plans and can assist orthodontists in analyzing and diagnosing image examinations. Additionally, it can monitor patient treatment progress and provide timely reminders for patient evaluations. Thus, the application of LLMs in orthodontics has significant potential and deserves in-depth research and exploration.

This scoping review intends to systematically collate the currently published literature on the application of LLMs in orthodontics to provide a comprehensive overview covering the applications, advantages, and challenges of LLMs in orthodontics. Through identifying gaps in current research, this review also aims to provide potential directions for future research and clinical practice.

## 2. Materials and Methods

In this scoping review, we performed a comprehensive review and synthesis of the published literature following the PRISMA-ScR guidelines [43] to ensure transparency and rationality in the whole process. Our research question was as follows: What are the applications of LLMs in orthodontics so far? All studies related to implementing LLMs in orthodontics were included in this review. The search strategy was structured based on “orthodontics” and “LLMs”, together with their synonyms (Appendix A). The eligibility criteria were constructed according to the PICOS format [44].
P (Population):Human subjects including patients, dental professionals, or laypeopleI (Intervention):LLMs, such as ChatGPT (Open AI), Gemini (Google), and Copilot (Microsoft), that were implemented in orthodontic domainsC (Comparator):Conventional healthcare approach or blank controlO (Outcomes):Assessment of the orthodontic outcomes in terms of diagnostic accuracy, treatment efficacy, speed of action, etc.S (Study design):Original studies published in English within peer-reviewed academic journals between 2017 and 30 June 2024 were included.

After an extensive search was performed across five major electronic databases (PubMed, Scopus, Web of Science, ProQuest, Embase), all records were imported into EndNote 20 software for screening. Initially, all duplicate literature records were removed. Then, two authors independently screened the title and abstract of each study based on the eligibility criteria. For those studies that met the eligibility criteria or where there was uncertainty, full text was retrieved for detailed evaluation. Any discrepancies that occurred during the screening process were resolved by discussion until consensus was reached.

The data extraction was independently conducted by two reviewers and pooled for analysis. The extracted data included characteristics of articles (authors, country, and publication year), LLMs (name and company), as well as the application (the performance/effectiveness) of the studies. This information was organized into a summary table of the included studies.

Eventually, we carried out a comprehensive analysis of the extracted data. A descriptive analysis of the collected basic information was performed, detailing the current state of published research from various aspects such as publication time, country, and predicting future research trends. Subsequently, we analyzed the research topics in-depth, discussing the specific applications of LLMs in different areas of orthodontics. This includes an assessment of current use, as well as an exploration of applications that may have potential in the future. Finally, we provided a detailed description and discussion of the challenges in the study, aiming to offer valuable insights for future research and applications.

## 3. Results

**Figure 1:**
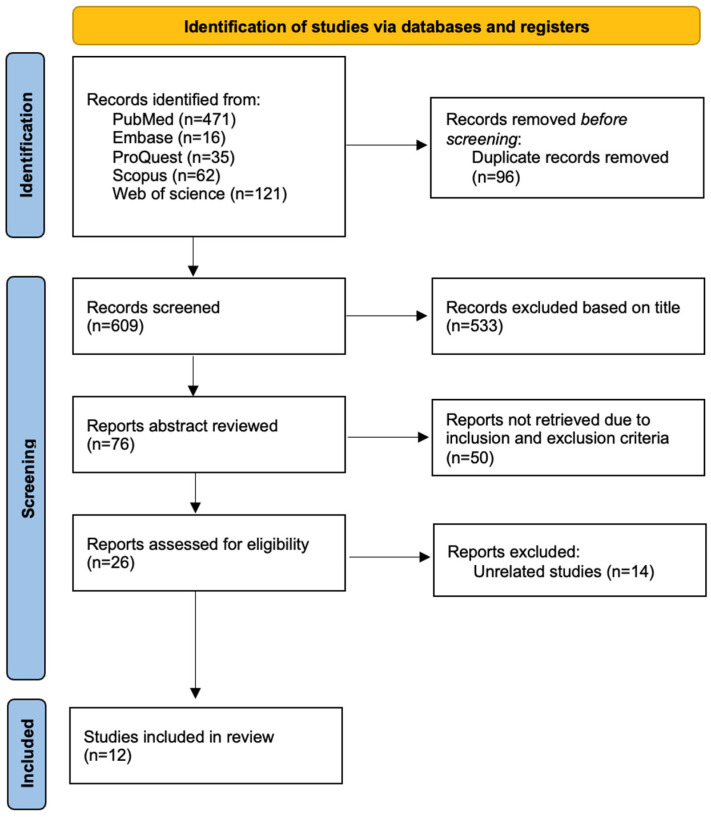
PRISMA-ScR flowchart.

The search obtained a total of 706 articles including 472 articles from PubMed, 16 articles from Embase, 35 articles from ProQuest, 62 articles from Scopus, and 121 articles from Web of Science. After the removal of 96 duplicate articles, 533 articles were excluded based on title screening. Following the abstract review, 50 articles did not meet the inclusion criteria, leaving 26 for full-text evaluation. Ultimately, only 12 articles fulfilled the eligibility criteria. The detailed flowchart is presented in Figure 1. The characteristics of the included studies are shown in Table 1.

**Figure 2:**
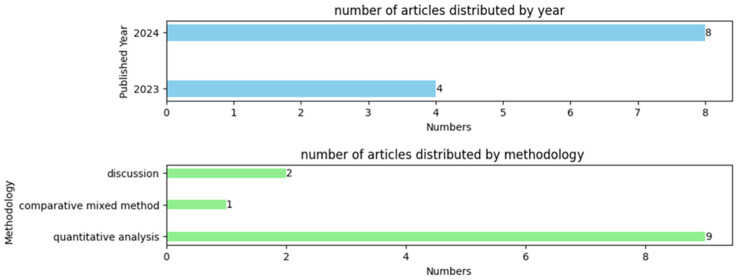
Number of articles distributed by year and methodology.

Of the 12 articles that met the inclusion criteria, all were published within the past two years (4 studies in 2023 and 8 studies in 2024); 9/12 (75%) articles evaluated the accuracy and validity of the different LLMs regarding the answers to questions related to the field of orthodontics through quantitative analysis; 1/12 (8.33%) article used the comparative mixed method and 2/12 (16.67%) articles discussed the application of LLMs. The distribution of these articles is illustrated in Figure 2.

**Figure 3:**
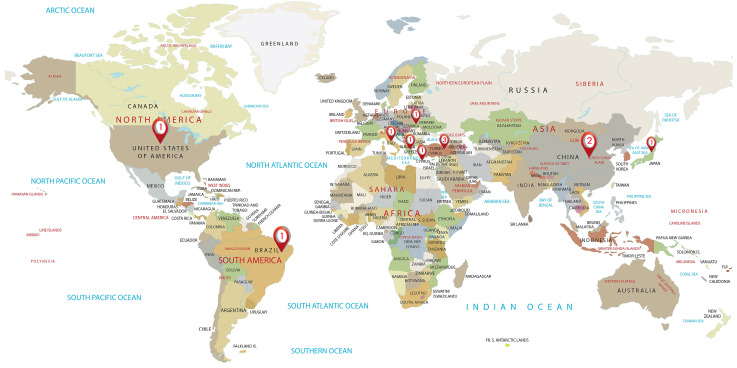
Country distribution of studies on the use of large language models.

Based on the regional distribution of these 12 articles, it can be seen that they were published across various countries. Specifically, the distribution includes three articles from Turkey, and one article each from Greece, Italy, the United States, Brazil, Slovakia, and Cyprus. The Asian region includes two articles from China and one from Japan. The detailed distribution of these articles is illustrated in Figure 3 (source: http://gisgeography.com). While this broad geographic distribution reflects the high level of interest among scholars worldwide in the application of LLMs in orthodontics, it also implies a relatively limited amount of research in this area.

The included articles were categorized based on the research methodology, and the majority of them are quantitative studies. Through statistical analyses, nine articles evaluated the effectiveness and accuracy of different LLMs in orthodontics. Four of these studies [48,51,52,53] demonstrate that LLMs perform well in providing accurate answers and effective assistance. However, five other articles [49,50,54,55,56] shows that the accuracy of these LLMs is not yet optimal, and they occasionally generate incorrect answers. Moreover, two discussion studies [46,47] presented the specific application of LLMs in DM software (Dental Monitoring Co., Paris, France) and the CephGPT-4 model, respectively, noting that they have enhanced the efficiency of orthodontic clinical treatment, but further research and development of these technologies are still needed. Furthermore, a mixed-method study [45] combining quantitative and qualitative approaches conducted a comprehensive assessment of the accuracy of responses provided by LLMs, with the results indicating that their overall accuracy needs improvement.

Additionally, twelve articles mentioned the application of different LLMs related to orthodontics. Surovková et al. [57] discussed how DM software can facilitate remote communication and monitoring between patients and dentists, noting its potential to provide personalized real-time analysis and feedback to help patients better understand the treatment process and enhancing treatment efficiency. L. Ma et al. [46] discussed the CephGPT4.0 model, which is trained based on MiniGPT-4 to generate diagnostic reports by automatically analyzing cephalometric landmarks. And there are two articles [45,55] that compared the performance of four LLMs (Bard, ChatGPT3.5, ChatGPT4.0, and Bing) in response to orthodontic questions; both articles showed that the LLMs occasionally generated suboptimal answers. Also, the study conducted by S. Abu Arqub et al. [49] highlighted that the overall accuracy of ChatGPT-3.5 cornering clear aligners was limited by a deficiency in relevant citations. But two other articles [48,53] demonstrated that ChatGPT4.0 exhibits a high level of accuracy and completeness in answering orthodontic questions. Furthermore, two articles [50,51] compared the accuracy of ChatGPT3.5 and Bard; it was found that both articles indicate that the quality of ChatGPT-3.5 is superior to Bard. Another two articles [52,54] compared the performance of ChatGPT3.5 and ChatGPT4.0; both indicate that ChatGPT-4.0 has improved in the reliability of responses but become more complex in terms of readability. Apart from these studies, M. Morishita et al. [56] evaluated the capabilities of ChatGPT4V, with a particular focus on its performance in processing image inputs and providing orthodontic analysis; however, the overall accuracy response rate was only 35% in responding to image-based questions.

Overall, we classified and discussed them according to different publication dates, geographical country distribution, and the application of different LLMs in various fields of orthodontics. It not only reveals the various applications and evaluations of LLM techniques, but also highlights their growing influence in the specialized field. For specific classifications and the studies, refer to Table 2.

## 4. Discussion

This rapid development of LLMs has created a revolution in the field of artificial intelligence, enabling them to be widely used in a variety of fields such as communication, education and healthcare [26,58,59,60]. LLMs, as an important part of artificial intelligence, are trained with large text data and deep learning techniques that enable them to learn and understand the patterns and structures of human language [61,62,63,64]. This ability makes LLMs display great potential in clinical, scientific research and other areas. In the medical field, LLMs can be applied to assist in diagnosis and personalized treatment planning, which can effectively improve the efficiency and quality of medical services [65,66,67].

With this scoping review, a total of 12 articles referring to the application of LLMs in orthodontics were collected, all of which were published during the last two years [45,46,47,48,49,50,51,52,53,54,55,56]. Based on our results, there are few studies on LLMs in orthodontics and a limited number of applied studies. It was also found that most of the articles evaluated the accuracy and speed of answering of different GPT models [45,50,51,52,54,55]. In addition, the included studies show an upward trend of research in this field over the years and these studies are conducted by researchers from different countries around the globe. The increasing number of studies from diverse regions in the past two years reflects the growing global attention and interest this field has attracted among scholars. However, our scoping review has some limitations. Firstly, it was restricted to English-language literature, and only five databases were searched, which introduces selection bias and limits the comprehensiveness of the evidence provided. So it indicates that there is still plenty of scope for further exploration and research in this area.

As the potential benefits of LLMs in various healthcare systems and cultural contexts become increasingly recognized, this provides strong support for the future development and application of LLMs in the field of orthodontics. For example, models based on the GPT architecture can analyze extensive datasets from the electronic health records (EHRs) of orthodontic patients to predict patient outcomes after treatment. This prediction ability not only helps dentists in formulating personalized treatment plans but also facilitates the early identification of potential risks, thereby enhancing patient prognosis [36,68,69]. Furthermore, LLMs can incorporate multimodal models, such as image recognition models, which leverage text and image data types to assist dentists in providing more comprehensive and accurate diagnoses. In the study by Morishita et al. [56], the image recognition capabilities and question-answering accuracy of ChatGPT-4V were evaluated, revealing that challenges still exist in processing complex images and providing accurate answers. In the future, more advanced machine learning models can be trained to improve image processing capabilities further, thereby providing better services to dentists and patients. Since some studies have shown that malocclusion may affect pronunciation [70,71,72], the combination of LLMs and specialized speech analysis models can assist in identifying potential risks of oral problems such as open bite and deep overbite, by analyzing speech data. Using this preliminary screening approach leads to further clinical examination and diagnosis; with this early detection and timely orthodontic intervention, it can effectively improve the oral health and quality of life of patients. Furthermore, LLMs show great potential in the field of patient care. Surovková et al. [47] have introduced and discussed the changes that AI technology can bring to doctors, nurses, and patients during orthodontic practice. Actually, in daily medical practice, patients often have questions about diagnostic reports and treatment plans. LLMs can assist healthcare staff in effectively conveying complex medical knowledge, helping patients better understand their treatment plans and increasing their compliance with treatment [73]. It also can help patients better understand their health conditions and enhance their health awareness by providing personalized educational content and resources. In addition, integrating the LLMs with general risk models can assist in triaging of orthodontic patients. By synthesizing and analyzing multiple data sources, LLMs can provide precise risk assessments. This application can optimize the allocation of medical resources by categorizing patients based on the time required for their visits, thus enabling more rational resource distribution and improving overall treatment efficiency and effectiveness [74]. LLMs also show distinct advantages in virtual simulated clinical trials [75]. Through creating virtual clinical trial environments, LLMs can evaluate the effects and risks of different orthodontic products and treatment plans. This not only accelerates the development of new products and therapies, but also provides a comprehensive analysis, thereby supporting researchers in making more informed decisions.

Additionally, in the field of education, LLMs play an important role in translating, explaining, summarizing, and synthesizing content [76,77]. They can break down complex content into multiple parts, making it easier for students to understand. At the same time, LLMs have the ability to guide students to gradually ask questions, promoting independent thinking and logical reasoning [78,79,80]. Furthermore, studies have shown that integrating LLMs into educational platforms can effectively optimize online teaching, improving the interactivity of learning and simulating clinical scenarios to train medical students [81]. By using LLMs to analyze and process a large amount of orthodontic case data, it can provide students with more accurate learning materials and simulated cases to help them gain a deeper understanding of complex orthodontic concepts and techniques, assisting them in fully understanding complex orthodontic concepts and techniques. Although the application of LLMs in orthodontic education can be regarded as an important direction for future research and development, continuous optimization and improvement are still needed to ensure the accuracy and reliability of the models and to avoid misinformation. Regardless of the challenges, the potential of LLMs in the field of education cannot be ignored. With the continuous development of technology and the expansion of application scenarios, LLMs are expected to play an important role in orthodontic education, promoting the innovation and development of educational models.

Even though the application of LLMs in the field of orthodontics can improve the accuracy of clinical diagnosis, assist in the personalized treatment plan making, and increase the efficiency of student learning, there are several limitations. First, it not only requires a large amount of high-quality training data to ensure the accuracy and reliability of generated information, but it is also necessary to establish a rapid and efficient data update mechanism to make the model capable of learning the latest medical developments in a timely manner. This is crucial because research and innovation in the medical field is continuous, and lacking up-to-date content may lead to inaccurate information. Secondly, LLMs present certain challenges in language understanding and generation. They may produce text that seems reasonable and coherent, but in reality, this content is not always accurate. In fields like medicine, where highly precise content is required, improving the reasoning and comprehension abilities of models becomes particularly crucial. Additionally, it is essential to take measures for ensuring patient data privacy and safety. In addition, continuous improvements must be made by verifying the accuracy and reliability of the outputs generated by LLMs.

## 5. Conclusions

In this scoping review, we present a thorough analysis of studies that explore the applications of LLMs in the field of orthodontics. Although the number of studies and enthusiasm for LLMs have gradually increased in recent years, there is still a gap in their application in the field of orthodontics. Existing research indicates that it is necessary to further improve the accuracy of the GPT in answering orthodontics-related questions, so that orthodontists can refer to the answers of the GPT to assist in formulating orthodontic treatment plans and also to help patients to better understand the questions in the diagnosis and treatment. It may be possible in the future to train the GPT with a large amount of data to improve its answer precision, and to develop relevant clinical applications based on LLMs that assist in treatment, which can significantly improve the treatment efficiency. For the education of dental students, applications based on LLMs can also be developed to enhance the efficiency and motivation of students. Issues related to privacy and ethics in artificial intelligence may be addressed in future research and development.

## Figures and Tables

**Table 1:** The characteristics of included studies.

Author and Year	Country	Objective	Assessment Method	Effects/Results
K. Giannakopoulos et al. (2023) [45]	Cyprus	To evaluate the accuracy of the answers provided by Bard, ChatGPT-3.5 and ChatGPT-4, and Bing Chat related to the two orthodontic questions. (1. Is early orthodontic treatment in two phases for children with prominent upper teeth more beneficial compared to treatment that is provided in one phase in adolescence? 2. Does orthodontic treatment affect airway function?)	Comparative Mixed Methods Study	Within the field of orthodontics, both quantitative and qualitative analyses showed that ChatGPT-4 and ChatGPT-3.5 perform significantly better than Google Bard and Microsoft Bing Chat.
L. Ma et al. (2023) [46]	China	In this paper, they propose a novel multimodal cephalometric analysis and diagnostic dialogue model called CephGPT-4.	Discussion	CephGPT-4 can improve the efficiency and accuracy of orthodontic measurements by automatically analyzing cephalometric landmarks and generating diagnostic reports but it still needs further validation and evaluation.
J. Surovková et al. (2023) [47]	Slovakia	The paper introduces the Dental Monitoring (DM), an orthodontic software that uses AI and knowledge-based algorithms to provide accurate treatment tracking and can answer questions from patients. It also evaluates DM’s clinical application within the daily workflow of orthodontic treatment.	Discussion	The use of DM can significantly improve treatment effectiveness by reducing both the average number of treatments and the overall duration, but it faces challenges with daily application imperfections and inconsistent scan evaluations. Although the use of LLMs in DM is limited, it can improve communication efficiency by semi-automatically answering questions from patients.
O. M. Tanaka et al. (2023) [48]	Brazil	To evaluate the accuracy of ChatGPT in answering to a total of 45 questions on Clear aligners, TAD and Digital imaging.	Quantitative analysis	225 evaluations of 5 evaluators show 11 (4.9%) were very poor, 4 (1.8%) as poor, and 15 (6.7%) as acceptable, good [34 (15,1%)] and very good [161 (71.6%)] (Fleiss’s Kappa = 0.004). ChatGPT has proven effective in providing quality answers related to these 3 domains.
S. Abu Arqub et al. (2024) [49]	USA	To assess the accuracy of ChatGPT answers of 111 questions concerning orthodontic clear aligners.	Quantitative analysis	ChatGPT provided correct responses to approximately 76% of the inquiries regarding orthodontic clear aligners but failed to provide the correct corresponding reference sources.
C. Arslan et al. (2024) [50]	Turkey	24 questions about conventional braces, clear aligners, orthognathic surgery and orthodontic retainers were chosen for assessing the accuracy of the answers provided by ChatGPT and BARD.	Quantitative analysis	Generally, these two provided satisfactory responses to the common orthodontic inquiries. ChatGPT’s answers surpassed those of Google Bard in quality. (The average number of references provided per answer: ChatGPT: 2.13 ± 1.51, BARD:1.96 ± 1.76).
B. Daraqel, et al. (2024) [51]	China	100 questions were used to evaluate and compare the performance of ChatGPT-3.5, Google Bard in terms of response accuracy, completeness, generation time, and response length when answering general orthodontic questions	Quantitative analysis	The median accuracy score was 9 (total score is 10) for ChatGPT and 8 for Bard. The median completeness score was 8 for ChatGPT and 8 for Bard. Bard’s response generation time was shorter than ChatGPT by 10.4 s/question. Response length generation was the same in the two models.
G. B. Demir et al. (2024) [52]	Turkey	To compare the effectiveness of ChatGPT3.5 and ChatGPT4 in completing systematic reviews.	Quantitative analysis	The accuracy rate of both PICO generation GPT4.0 was better than that of GPT3.5, especially in the P and C parts (initial accuracy rates: GPT-4: P = 98%,C = 86%; GPT-3.5: P = 93%, I = 63%; second GPT-4: P = 100%,C = 81%; GPT-3.5: P = 88%,C = 42%) Both ChatGPT 3.5 and 4 can be pivotal tools for generating PICO-driven queries in orthodontics.
A. Hatia et al. (2024) [53]	Italy	Twenty-one questions were used to investigate the accuracy and completeness of ChatGPT in answering questions and solving clinical scenarios related to interceptive orthodontics.	Quantitative analysis	For open-ended questions, the overall median score was 4.9/6 for the accuracy, and 2.4/3 for completeness. For clinical cases, the overall median score was 4.9/6 for the accuracy, and 2.5/3 for completeness.
D. D. Kılınç and D. Mansız (2024) [54]	Turkey	34 questions about orthodontics were used to assess the reliability and readability of the responses to the two versions of ChatGPT.	Quantitative analysis	ChatGPT’s responses showed some improvement in reliability aspects during the second evaluation (*p* = 0.001). The readability of the response texts in the new version became more difficult (*p* = 0.001).
M. A. Makrygiannakis et al. (2024) [55]	Greece	Ten questions about orthodontics were used to assess and compare the answers provided by Google’s Bard, OpenAI’s ChatGPT-3.5 and ChatGPT-4, and Microsoft’s Bing.	Quantitative analysis	Bing shows the highest answering score (Bing = 7.1, ChatGPT-4.0 = 4.7, Google Bard = 4.6, ChatGPT-3.5 = 3.8 the score ranging from 0 to 10) All models occasionally produced answers with a lack of comprehensiveness, accuracy, clarity and relevance.
M. Morishita et al.(2024) [56]	Japan	A total of 160 questions were used to assess the capabilities of ChatGPT-4V in answering image-based questions, including 20 questions specifically in the field of orthodontics.	Quantitative analysis	ChatGPT-4V has some limitations; the overall correct response rate of ChatGPT-4V was 35%, 57.1% for compulsory questions, 43.6% for general questions, 28.6% for practical questions. In the field of orthodontics, the correct answer rate was 25%. Of the 22 unanswered questions, 36.4% were orthodontics.

**Table 2:** Specific classifications and studies.

Published Year	Methodological	Region Distribution	LLMs	The Field of Orthodontics
2023 K. Giannakopoulos et al. (2023) [45] L. Ma et al. (2023) [46] J. Surovková et al. (2023) [47] O. M. Tanaka et al. (2023) [48] 2024 S. Abu Arqub et al. (2024) [49] C. Arslan et al. (2024) [50] B. Daraqel, et al. (2024) [51] G. B. Demir et al. (2024) [52] A. Hatia et al. (2024) [53] D. D. Kılınç and D. Mansız (2024) [54] M. A. Makrygiannakis et al. (2024) [55] M. Morishita et al.(2024) [56]	Discussion L. Ma et al. (2023) [46] J. Surovková et al. (2023) [47] Comparative mixed method K. Giannakopoulos et al. (2023) [45] Quantitive analysis O. M. Tanaka et al. (2023) [48] S. Abu Arqub et al. (2024) [49] C. Arslan et al. (2024) [50] B. Daraqel, et al. (2024) [51] G. B. Demir et al. (2024) [52] A. Hatia et al. (2024) [53] D. D. Kılınç and D. Mansız (2024) [54] M. A. Makrygiannakis et al. (2024) [55] M. Morishita et al.(2024) [56]	Europe K. Giannakopoulos et al. (2023) [45] J. Surovková et al. (2023) [47] C. Arslan et al. (2024) [50] G. B. Demir et al. (2024) [52] A. Hatia et al. (2024) [53] D. D. Kılınç and D. Mansız (2024) [54] M. A. Makrygiannakis et al. (2024) [55] Asia L. Ma et al. (2023) [46] B. Daraqel, et al. (2024) [51] M. Morishita et al.(2024) [56] South America O. M. Tanaka et al. (2023) [48] North America S. Abu Arqub et al. (2024) [49]	ChatGPT K. Giannakopoulos et al. (2023) [45] O. M. Tanaka et al. (2023) [48] S. Abu Arqub et al. (2024) [49] C. Arslan et al. (2024) [50] B. Daraqel, et al. (2024) [51] G. B. Demir et al. (2024) [52] A. Hatia et al. (2024) [53] D. D. Kılınç and D. Mansız (2024) [54] M. A. Makrygiannakis et al. (2024) [55] M. Morishita et al.(2024) [56] Bard K. Giannakopoulos et al. (2023) [45] C. Arslan et al. (2024) [50] B. Daraqel, et al. (2024) [51] M. A. Makrygiannakis et al. (2024) [55] Bing K. Giannakopoulos et al. (2023) [45] M. A. Makrygiannakis et al. (2024) [55] MiniGPT-4 L. Ma et al. (2023) [46]	Clinical Applications Assisting treatment and question answering K. Giannakopoulos et al. (2023) [45] L. Ma et al. (2023) [46] J. Surovková et al. (2023) [47] O. M. Tanaka et al. (2023) [48] S. Abu Arqub et al. (2024) [49] C. Arslan et al. (2024) [50] B. Daraqel, et al. (2024) [51] G. B. Demir et al. (2024) [52] A. Hatia et al. (2024) [53] D. D. Kılınç and D. Mansız (2024) [54] M. A. Makrygiannakis et al. (2024) [55] Automatic diagnosis and imagine analysis M. Morishita et al.(2024) [56]

## Data Availability

The original data presented in the study are openly available in the references.

## Appendix A

**Search strategy**

**PubMed: 471**

#1 ((((((((Large Language Model*) OR (LLM)) OR (ChatGPT)) OR (GPT)) OR (generative pre trained transformer*)) OR (pretraining language model*)) OR (AI language model)) OR (chatbot)) OR (gpt-*) AND (2017:2024[pdat])

#2 (((((((orthodon*) OR (cephalo*)) OR (orthodontic treatment)) OR (Dental braces)) OR (Aligners)) OR (Teeth straightening)) OR (Dental correction)) OR (orthodontic procedure) AND (2017:2024[pdat])

#1 AND #2

**Embase: 16**

#1 (‘large language model*’:ti,ab,kw OR llm:ti,ab,kw OR chatgpt:ti,ab,kw OR gpt:ti,ab,kw OR ‘generative pre trained transformer*’:ti,ab,kw OR ‘pretraining language model*’:ti,ab,kw OR ‘ai language model’:ti,ab,kw OR chatbot:ti,ab,kw OR gpt*:ti,ab,kw) AND [2017–2024]/py

#2 (orthodon*:ti,ab,kw OR cephalo*:ti,ab,kw OR ‘orthodontic treatment’:ti,ab,kw OR ‘dental braces’:ti,ab,kw OR aligners:ti,ab,kw OR ‘teeth straightening’:ti,ab,kw OR ‘dental correction’:ti,ab,kw OR ‘orthodontic procedure’:ti,ab,kw) AND [2017–2024]/py

#3 #1 AND #2

**ProQuest: 35**

#1 noft(Large Language Model*) OR noft(LLM) OR noft(ChatGPT) OR noft(GPT) OR noft(generative pre trained transformer*) OR noft(pretraining language model*) OR noft(AI language model) OR noft(chatbot) OR noft(gpt*)

#2 noft(orthodon*) OR noft(cephalo*) OR noft(orthodontic treatment) OR noft(Dental braces) OR noft(Aligners) OR noft(Teeth straightening) OR noft(Dental correction) OR noft(orthodontic procedure)

#3 #1 AND #2

**Scopus: 62**

#1 (TITLE-ABS-KEY (large AND language AND model*) OR TITLE-ABS-KEY (llm) OR TITLE-ABS-KEY (chatgpt) OR TITLE-ABS-KEY (gpt) OR TITLE-ABS-KEY (generative AND pre AND trained AND transformer*) OR TITLE-ABS-KEY (pretraining AND language AND model*) OR TITLE-ABS-KEY (ai AND language AND model) OR TITLE-ABS-KEY (chatbot) OR TITLE-ABS-KEY (gpt*)) AND PUBYEAR > 2016 AND PUBYEAR < 2025

#2 (TITLE-ABS-KEY (orthodon*) OR TITLE-ABS-KEY (cephalo*) OR TITLE-ABS-KEY (orthodontic AND treatment) OR TITLE-ABS-KEY (dental AND braces) OR TITLE-ABS-KEY (aligners) OR TITLE-ABS-KEY (teeth AND straightening) OR TITLE-ABS-KEY (dental AND correction) OR TITLE-ABS-KEY (orthodontic AND procedure)) AND PUBYEAR > 2016 AND PUBYEAR < 2025

#3 #1AND #2

**Web of science: 121**

#1 ((((((((TS=(Large Language Model*)) OR TS=(LLM)) OR TS=(ChatGPT)) OR TS=(GPT)) OR TS=(generative pre trained transformer*)) OR TS=(pretraining language model*)) OR TS=(AI language model)) OR TS=(chatbot)) OR TS=(gpt*)

#2 (((((((TS=(orthodon*)) OR TS=(cephalo*)) OR TS=(orthodontic treatment)) OR TS=(Dental braces)) OR TS=(Aligners)) OR TS=(Teeth straightening)) OR TS=(Dental correction)) OR TS=(orthodontic procedure)

#3 #1 AND #2
